# Exosomes and solid cancer therapy: where are we now?

**DOI:** 10.1007/s12032-025-02626-3

**Published:** 2025-02-17

**Authors:** Tomas Zemanek, Lubos Danisovic, Andreas Nicodemou

**Affiliations:** 1 Institute of Medical Biology, Genetics and Clinical Genetics, Faculty of Medicine, Comenius University, Bratislava, Slovakia; 2 GAMMA - ZA s.r.o., Trencin, Slovakia

**Keywords:** Exosomes, Cancer, Immunotherapy, Personalized medicine, Cell therapy, Drug delivery system

## Abstract

Cancer immunotherapy has revolutionized oncology, offering new hope for patients with previously incurable cancers. However, solid tumors remain a significant challenge due to immune evasion, therapeutic resistance, and the immunosuppressive tumor microenvironment. Exosomes, a specialized subset of extracellular vesicles, have emerged as promising tools in cancer therapy owing to their unique role in intercellular communication and immune modulation. These vesicles transport antigens, major histocompatibility complex (MHC) molecules, and immune-modulatory cargo, positioning them as potential platforms for cancer vaccines, drug delivery systems, and combinatorial therapies. Advances in engineered exosomes have improved drug bioavailability, tumor targeting, and immune stimulation, showcasing their potential in personalized medicine. This review highlights their multifaceted role in the tumor microenvironment, and their mechanisms of action in solid cancer therapy. Additionally, we discuss emerging strategies to overcome clinical and technical hurdles, paving the way for novel and effective cancer treatments.

## Introduction

Cancer immunotherapy has revolutionized oncology, particularly in hematologic malignancies, yet its efficacy in solid tumors remains limited. Major challenges include poor immune infiltration, an immunosuppressive tumor microenvironment (TME), and physical barriers imposed by the extracellular matrix (ECM), all of which restrict immune cell access and drug delivery [1, 2]. The TME, comprising tumor cells, cancer-associated fibroblasts (CAFs), immune cells, and stromal components, fosters immune evasion and therapeutic resistance [3].

**Fig. 1:**
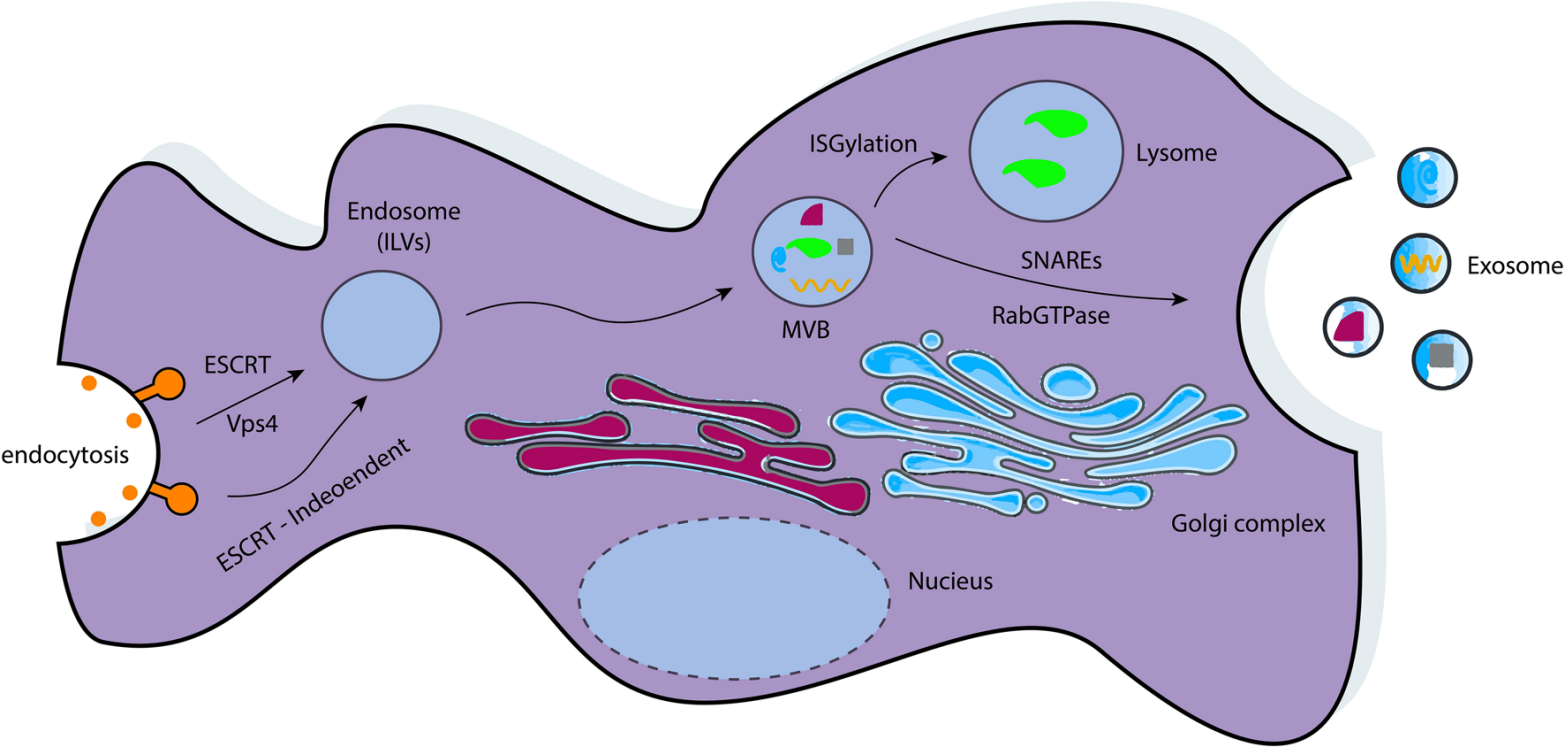
Exosome biogenesis. Illustration of the cellular mechanisms of exosome biogenesis. Endocytosis results in the formation of early endosomes, which mature into multivesicular bodies (MVBs) containing intraluminal vesicles (ILVs). Exosome formation can involve ESCRT-dependent or ESCRT-independent pathways, with the crucial ATPase Vps4 disassembling the ESCRT complex to facilitate vesicle formation. MVBs either fuse with lysosomes for degradation or with the plasma membrane to release exosomes. Regulatory proteins like Rab GTPases and SNAREs facilitate vesicular transport. Exosomes carry diverse biomolecules (e.g., proteins, RNA) and play crucial roles in intercellular communication

Extracellular vesicles (EVs), particularly exosomes, have emerged as key mediators of cell-to-cell communication in the TME. Exosomes, primarily formed through multivesicular body fusion with the plasma membrane [4], transport biomolecules—including nucleic acids, proteins, and lipids—that influence immune regulation and tumor progression [5]. Their ability to cross biological barriers, such as the blood–brain barrier (BBB), further underscores their therapeutic potential [6]. Exosome biogenesis has been described elsewhere [7]. Their biogenesis (Fig. 1), cargo composition, and role in the TME are integral to their therapeutic potential. These aspects are discussed in detail in next sections.

Recent advances highlight exosomes as promising tools in cancer immunotherapy. Engineered exosomes can deliver tumor antigens and immune checkpoint inhibitors, enhancing antigen presentation and immune activation [8]. Dendritic cell-derived exosomes (DEXs) and chimeric antigen receptor T (CAR-T) exosomes have shown the ability to modulate the TME, promoting immune infiltration and reducing immunosuppression [9]. Tumor-derived exosomes (TDEs) are being explored as both therapeutic targets and delivery systems, though their dual role in cancer progression remains a challenge [10].

This review examines the role of exosomes in the TME, their therapeutic applications, and recent advances in exosome-based immunotherapies. Additionally, we discuss clinical applications and the challenges that must be addressed for their successful translation into solid cancer treatment.

## Sources of exosomes in human medicine

Mammalian cells as reticulocytes, platelets, T and B cells, macrophages, neurons, stem cells, body fluids and also tumor cells represent great source of exosomes and have been approved for use in clinical settings and investigated as possible therapies, furthermore, there has been reports of successful utilization of plant and milk-derived exosomes as well [11–13].

## Mechanisms of exosomal uptake and implications in cancer therapy

After exosomes are released into the extracellular space, they interact with recipient cells through various mechanisms, influenced by factors such as the cell types involved and the molecular composition of both the exosome and recipient cell surfaces. While the exact mechanisms underlying exosome targeting remain poorly understood, it is unclear whether exosome delivery is random or specific to certain destinations [14]. Upon reaching a recipient cell, exosomes can be internalized via endocytosis, direct fusion with the plasma membrane, or interaction with cell-surface receptors, triggering intracellular signaling pathways [15]. Endocytosis, where cells engulf extracellular material via the plasma membrane, is the predominant mechanism for exosome uptake. Exosomes may enter cells through clathrin-mediated endocytosis, caveolin-mediated endocytosis, macropinocytosis, or phagocytosis [16]. Following endocytosis, exosomes can fuse with the endosome membrane, releasing their contents into the cytoplasm. Alternatively, exosomes can bypass endocytosis and directly fuse with the recipient cell’s plasma membrane, analogous to viral envelope fusion during host cell entry [17]. This direct fusion route is considered more effective for delivering cargo into the cytoplasm [18].

To exert effects within recipient cells, exosomal cargo must escape from endosomes into the cytoplasm through a process known as endosomal escape. Without it, cargo may either be degraded by lysosomes or expelled back into the extracellular space, reducing its therapeutic potential [19, 20]. This highlights the appeal of the direct fusion route for exosome-based drug delivery systems. The cargo, including proteins, lipids, RNA, and drugs, can modulate recipient cell functions by altering gene expression, modifying signaling pathways, or inducing immune responses. Exosome surface proteins also play a role in cellular interactions. Molecules such as fibronectin, tetraspanins, immunoglobulins, proteoglycans, lectin receptors, syncytin-1, and syncytin-2 are known to mediate exosome-cell interactions [21–23]. Notably, exosomal ligands like PD-L1, TNF, FasL, and TRAIL, which bind to receptors on tumor cells, are being explored as potential tools for cancer therapy. For instance, exosomes with MHC-peptide complexes can directly activate immune cells, while exosomes from prostate cancer cells carrying PD-L1 bind to PD-1 on T cells, suppressing their activation [24]. Similarly, exosomes mediate the transfer of chemokine receptor CCR5 between monocytes and endothelial cells, playing a role in HIV-1 transmission [25]. In the nervous system, exosomes facilitate protein delivery, such as synaptotagmin 4, from presynaptic to postsynaptic cells [26].

The specificity of exosome uptake is influenced by both donor and recipient cell types. For instance, neuroblastoma cell-derived exosomes are taken up by both neurons and glial cells, whereas exosomes from cortical neurons are exclusively captured by other neurons [27]. Similarly, lung cancer A549 cell-derived exosomes can be internalized by colorectal cancer HCT116 cells and vice versa, suggesting some degree of non-specific uptake [28]. Targeting efficiency can, however, be enhanced by specific ligands, such as GE11 peptides on exosomes, which bind to EGFR on tumor cells and promote selective uptake [29]. Additionally, CD47 expression on exosomes helps them evade macrophage-mediated phagocytosis, increasing their likelihood of being absorbed by recipient cells [30]. These findings underscore the complex and multifaceted nature of exosomal uptake, which remains an active area of investigation. Despite recent advances, variability in exosome uptake across different cell types poses a significant challenge for clinical applications in exosome-based drug delivery systems [31]. Nonetheless, the ability of exosomes to efficiently transport therapeutic agents into cells has fueled growing interest in their potential in this research field [32].

## Exosomes in cancer therapy – current progress

Recent clinical trials are increasingly investigating exosome-based therapies for solid cancers, focusing on drug delivery, immunotherapy, and diagnostics. These trials target a broad spectrum of malignancies, including lung, breast, pancreatic, colorectal, ovarian, and glioma, as well as more challenging cancers such as hepatocellular carcinoma and cholangiocarcinoma.

As summarized in Table 1, therapeutic approaches range from exosome-mediated drug delivery such as paclitaxel-loaded exosomes to immunotherapy using dendritic cell-derived exosomes and RNA-based interventions like siRNA or mRNA delivery. Some trials also explore combinatorial strategies, integrating exosomes with immune checkpoint inhibitors or chemotherapy. Beyond treatment, exosome-based liquid biopsy assays and predictive biomarkers are being developed to enhance early cancer detection and treatment response monitoring. For a concise overview of key exosome characteristics discussed in this chapter, please refer to Table 2. Since the ongoing clinical trials summarized in Table 1 provide limited data, we have also included Table 3, which highlights successful applications of exosome-based therapies in cancer and can be found at the end of the section.

**Table 1:** Application of exosomes in ongoing clinical trials of solid cancer (Data source: ClinicalTrials.gov: https://clinicaltrials.gov/ accessed in Feb 2025)

Clinical trial identifier	Phase	Cancer type	Source of exosomes	Cargo	Therapy
NCT04427475	N/A	Non-Small Cell Lung Carcinoma	–	–	Pabolizumab, Nafulizumab
NCT05427227	N/A	Advanced/Late-Stage Gastrointestinal Cancer	–	–	Immunotherapy, anti-HER2 therapy, anti-CLDN18.2 therapy
NCT05575622	N/A	Hepatocellular Carcinoma	–	–	Immunotherapy
NCT05705583	N/A	Renal Cell Carcinoma	–	–	Immune Checkpoint Inhibitors (ICIs)
NCT05955521	N/A	HER2-positive Breast Cancer, Triple-Negative Breast Cancer	–	–	Neoadjuvant Chemotherapy
NCT06388967	N/A	Pancreatic Cancer	–	–	PANXEON Diagnostic Test
NCT06536712	I	Rectal Cancer	Mesenchymal Stem Cells	Exosomes	MSC-derived Exosome Therapy
NCT06558019	N/A	Ovarian Cancer	–	–	Exosome-based OCS Scores
NCT06278064	N/A	Esophageal Cancer, Gastric Cancer	–	–	–
NCT01294072	N/A	Colon Cancer	Plant-derived Exosomes	Curcumin, Curcumin-conjugated Exosomes	Curcumin Therapy
NCT04939324	N/A	Non-Small Cell Lung Carcinoma, Lung Cancer	–	–	Surgery
NCT06116903	N/A	Glioma	–	–	Chemotherapy, Radiotherapy
NCT02393703	N/A	Pancreatic Cancer	–	–	Surgery
NCT05463107	N/A	Thyroid Cancer	–	–	Surgery
NCT05286684	N/A	Breast Cancer	–	–	–
NCT06342414	N/A	Hepatic Cancer	–	–	ELUCIDATE Diagnostic Panel
NCT03608631	I	Pancreatic Cancer	Mesenchymal Stromal Cells	KRAS G12D siRNA	MSC-derived Exosomes Therapy
NCT02147418	N/A	Oropharyngeal Cancer	–	–	–
NCT03108677	N/A	Lung Metastases, Osteosarcoma	–	–	–
NCT06342401	N/A	Colorectal Cancer	–	–	ENCODE Diagnostic Test
NCT04948437	N/A	Thyroid Cancer	–	–	Surgery
NCT03824275	II/III	Prostatic Neoplasms	–	–	18F-DCFPyL PET/CT
NCT05270174	N/A	Bladder Cancer, Lymphatic Metastasis	–	lncRNA-ELNAT1	–
NCT06381648	N/A	Cholangiocarcinoma	–	–	LyMIC Diagnostic Test
NCT05744076	N/A	Melanoma	–	–	Immunotherapy
NCT04288141	N/A	HER2-positive Breast Cancer	–	–	HER2-directed Therapy
NCT05587114	N/A	Lung Cancer	–	–	-
NCT06342440	N/A	Colorectal Cancer	–	–	DENEB Diagnostic Test
NCT03711890	N/A	Pancreatic Carcinoma	–	–	OCT + Surgery
NCT05625529	N/A	Pancreatic Cancer	–	–	ExoVerita™ Assay
NCT04499794	N/A	Advanced NSCLC Patients	–	–	ALK Inhibitor
NCT05218759	N/A	Non-Small Cell Lung Cancer	–	–	Anlotinib
NCT06026735	N/A	Lung Cancer with CNS Metastasis	–	–	–
NCT04629079	N/A	Lung Cancer	–	–	–
NCT04852653	N/A	Rectal Cancer	–	–	Chemotherapy, Radiotherapy
NCT06245746	I	Acute Myeloid Leukemia	Umbilical Cord Mesenchymal Stem Cells (UCMSC)	Exosomes	UCMSC-Exo Therapy
NCT03800121	N/A	Sarcoma	–	–	Neoadjuvant Chemotherapy
NCT04053855	N/A	Clear Cell Renal Cell Carcinoma	–	–	Surgery
NCT03985696	N/A	Aggressive Non-Hodgkin Lymphoma	–	–	–
NCT05705583	N/A	Renal Cell Carcinoma	–	–	–
NCT05427227	N/A	Advanced/Late-Stage Gastrointestinal Cancer	–	–	Immunotherapy, anti-HER2 therapy, anti-CLDN18.2 therapy

**Table 2:** Summary of key findings on exosomes in cancer immunotherapy

Category	Key Findings	Therapeutic Potential
Engineered Exosomes in Antitumor Immunity	• Dendritic cell-derived exosomes (DEXs) engineered with HCC-targeting peptide, immunoadjuvants, and tumor epitopes elicit immune responses • Exosomes modified with α-LA deliver immunogenic agents to TNBC models • Exosomes with dual antibodies (CD3 & HER2) enhance T cell recruitment and cancer cell targeting • EVs from lung cancer cells deliver oncolytic viruses & chemotherapy agents	• Amplify immunotherapy efficacy • Transform immunologically "cold" tumors into "hot" tumors for better immune recognition
Exosome-Mediated siRNA Therapy	• EVs deliver PD-L1 siRNA to glioblastoma, improving immune response • Combined oxaliplatin & galectin-9 siRNA delivery induces immunogenic cell death and macrophage repolarization • CD47-expressing exosomes fused with liposomes deliver GM-CSF & docetaxel, promoting M1 macrophage polarization	• Enhances immune checkpoint therapy in resistant cancers • Overcomes immunosuppressive tumor microenvironments (TMEs)
Dendritic Cell-Derived Exosomes (Dexs)	• Dexs activate DCs in TNBC & glioblastoma models • Universal Dex nano-vaccine strategy developed for HCC • Dex-based therapies show high response rates and safety in clinical trials	• Personalized cancer therapy potential • Needs optimization for scalability & purification
T Cell-Derived Exosomes	• CD4^+^ EVs enhance CD8^+^ T cell cytotoxicity & macrophage activation • Vδ2-T cell exosomes induce apoptosis in EBV-associated gastric carcinoma • CAR-T derived exosomes carrying paclitaxel target lung tumors efficiently	• Supports immune regulation & cancer cell targeting • Overcomes conventional CAR-T therapy limitations
B Cell-Derived Exosomes	• Display monomeric IgM and antigen-specific IgG • Provide antigen-specific protection in influenza models • Can trigger autoimmune responses under certain conditions	• Offer an independent antibody delivery system • May be used for immune system modulation
NK Cell-Derived Exosomes	• Let-7b-5p miRNA from NK exosomes inhibits pancreatic cancer proliferation • NK exosomes loaded with miR-30c enhance TNF-α and IFN-γ levels, boosting cytotoxicity • CAR-NK exosomes combined with nanomedicine (ExoCAR/T7@Micelle) cross the BBB & target HER2+ breast cancer metastases	• Effective in direct tumor targeting • Non-toxic, with promising applications in metastatic cancers
CAF-Derived Exosomes	• CAF reprogramming strategy developed for pancreatic cancer drug delivery • CD9/CD63^+^ CAF exosomes reduce myeloma proliferation & improve patient survival	• Transform tumor-supporting fibroblasts into therapeutic tools
Tumor-Derived Exosomes (TDEs)	• Promote metastasis, immune suppression, and pre-metastatic niche formation • Engineered TDEs can carry tumor antigens for vaccine development • Can be modified to enhance immunostimulatory properties	• Dual role in cancer progression and therapy • Requires careful engineering to avoid tumor promotion

**Table 3:** Exosome-based cancer treatments and outcomes

Cancer type	Application	Key findings	Reference
Glioma	miR-146b (MSCs)	Inhibited glioma cell growth Inhibited tumor growth	[33]
Breast Cancer	Let-7a miRNA (Embryonic kidney cells)	Inhibited tumor growth	[34]
	miR-379 (MSCs)	Increased tumor necrosis	[35]
	Cas9 mRNA (RBCs)	Inhibited breast cancer cell growth, Inhibited tumor growth	[36]
	MHC-I/peptide complexes (Dendritic cells)	Increased T cell response	[37]
	Ex-DC@CQDs	Enhanced tumor targeting and therapeutic efficacy	[38]
	miRNA-159 & doxorubicin	Enhanced drug uptake Demonstrated synergistic therapeutic effects both in vitro and in vivo	[39]
	3,3′-diindolylmethane & doxorubicin	Effectively attenuated cancer stem cell-driven epithelial-mesenchymal transition (EMT)	[40]
Hepatocellular Carcinoma (HCC)	miR-335 − 5p (Stellate cells)	Inhibited HCC cells growth and invasion Inhibited tumor growth	[41]
	Exosomal miR-122	Enhanced chemosensitivity of HCC	[42]
	miR-320a	Effectively inhibited HCC proliferation and metastasis	[43]
Pancreatic Cancer	miR-145 − 5p (MSCs)	Inhibited PDAC cell proliferation and invasion, Increased tumor apoptosis	[44]
	KrasG12D siRNA (Fibroblast-like mesenchymal cells)	Increased apoptosis of Panc-1 cell Decreased tumor size	[30]
	Curcumin	Apoptosis of pancreatic cancer cell	[45]
	dtEVs	Effectively suppressed large solid PDAC	[46]
Colorectal Cancer	miR-25 − 3p inhibitor	Inhibited tube formation of HUVEC cell Inhibited formation of premetastatic niche	[47]
	Target-Her2-LAMP2-GFP, THLG-Exo/5-FU/miR-21i	Effectively reversed drug resistance in colorectal cancer cells	[48]
Tongue Carcinoma	ECRG4 mRNA (Serum)	Inhibited tongue carcinoma cell growth	[49]
Neuroblastoma	Hsp27 siRNA	Inhibited differentiation of neuroblastoma cells	[50]
Lymphoma	TRAIL (Myeloid leukemia cells)	Increased apoptosis of leukemia cells, Did not affect tumor growth	[51]
	Dual-targeting exosomes	Effectively slowed tumor progression across various animal tumor models, surpassing traditional tumor vaccines and T cell reinfusion therapies	[52]
Epidermal Carcinoma	EGFR nanobodies (Mouse neuroblast cells)	Inhibited epidermal carcinoma cell growth	[53]
Colon Cancer	Competitive antagonist (SIRPα) (Embryonic kidney cells)	Increased phagocytosis of macrophages	[54]
Ovarian Cancer	Cisplatin (Hepatocarcinoma & ovarian cancer cells)	Inhibited ovarian cancer & hepatocarcinoma cell growth Inhibited tumor growth	[55]
	MsEV-siYTHDF1-DTX	Significantly improved tumor inhibition and extended survival in tumor-bearing mice	[56]
Lewis Lung Carcinoma	Paclitaxel (Macrophages)	Inhibited Lewis Lung Carcinoma cell proliferation, Inhibited tumor growth	[57]
Prostate Cancer	Paclitaxel (Prostate cancer cells)	Inhibited prostate cancer cell growth	[58]
Chronic Myelogenous Leukemia	Modified exosomes containing IL3-Lamp2B, loaded with Imatinib	Reduced tumor size	[59]
Multiple Myeloma (MM)	HSP70 (Myeloma cell)	Increased T cell response	[60]
	BTZ/PC-apoVs	Significantly increased apoptosis in MM cells, stronger antitumor activity	[61]
Glioblastoma	iExo-Myc	Significantly inhibited tumor proliferation and angiogenesis	[62]
Renal Cell Carcinoma (RCC)	miRNA-1	Inhibited RCC growth and invasion	[63]
	Exosomes containing GPI-IL-12	Notable cytotoxic effects	[64]
	Exosomal circSPIRE1	Inhibited both angiogenesis and vessel permeability	[65]
Malignant Melanoma	cRGD-Exo/TP	Prolonged circulation time, higher tumor accumulation, better targeting	[66]
Osteosarcoma	EM-Dox	Enhanced drug delivery specificity, minimized off-target toxicity	[67]
Lung Cancer	FA-ExoPAC	Improved drug delivery efficiency, significantly reduced toxicity	[68]
Gastric Cancer	miR-21 inhibitors	Increased inhibitory effects, reduced cytotoxicity	[69]
	DC-derived exosome vaccines	Enhanced T cell immune response, tumor rejection	[70]
	ExoDOX	Reduced cardiotoxicity	[71]

### Engineered exosomes in antitumor immunity and immune modulation

Engineered exosomes have emerged as promising tools in antitumor immunity. Derived from parental cells, dendritic cell-derived exosomes (DEXs) carry abundant signature proteins and have been explored as innovative vaccine candidates [72]. For instance, engineered DEXs with hepatocellular carcinoma (HCC)-targeting peptide P47, the immunoadjuvant high-mobility group nucleosome-binding protein 1 (HMGN1), and an α-fetoprotein epitope. This vaccine system effectively recruited and activated dendritic cells at tumor sites, eliciting antigen-specific immune responses and inducing tumor-killing effects in orthotopic HCC mouse models [8]. Similarly, exosomes modified with α-lactalbumin (α-LA) delivered human neutrophil elastase (ELANE) and the Toll-like receptor 3 (TLR3) agonist Hiltonol, both inducers of immunogenic cell death, to triple-negative breast cancer (TNBC) models. This approach enhanced CD8^+^ T cell responses in mouse xenografts and patient-derived tumor organoids. Additionally, exosomes with dual antibodies targeting CD3 T cells and HER2 receptors facilitated T cell recruitment and specific killing of HER2-positive breast cancer cells [9]. Lung cancer cell-derived extracellular vesicles (EVs) demonstrated the ability to deliver oncolytic viruses (OVs) and chemotherapy agents, such as paclitaxel, to tumor sites, leading to enhanced antitumor effects in nude mouse models [73]. These studies highlight exosomes’ potential as delivery platforms to amplify the efficacy of immunotherapeutic agents and transform immunologically "cold" tumors into "hot" tumors.

Exosome-mediated delivery of small interfering RNAs (siRNAs) targeting critical immune-related genes also represents a promising avenue in antitumor therapy. While immune checkpoint blockade has shown efficacy in certain cancer types, its success in glioblastoma (GBM) remains limited due to the blood‒brain barrier (BBB) and the suppressive tumor microenvironment [74]. It was also demonstrated that C(RGDyK)-conjugated EVs could effectively deliver PD-L1 siRNA to GBM cells, alleviating the immunosuppressive microenvironment following short-burst radiation, thereby offering a viable strategy for immune checkpoint therapy in brain tumors [75]. Likewise, the combined delivery of oxaliplatin and siRNA targeting galectin-9 triggered immunogenic cell death and reversed M2 tumor-associated macrophage-mediated immunosuppression in pancreatic ductal adenocarcinoma [76]. Exosomes also hold significant potential in treating metastatic cancers due to their targeting and penetrating capabilities. For example, a nano delivery system by fusing CD47-expressing exosomes with thermosensitive liposomes loaded with granulocyte–macrophage colony-stimulating factor (GM-CSF) and docetaxel. This system promoted macrophage repolarization toward the M1 phenotype, achieving superior therapeutic outcomes compared to either agent alone in models of metastatic peritoneal carcinoma [77].

### Dendritic cell-derived exosomes

Dendritic cells (DCs), belonging to antigen-presenting cells (APCs) carry out the induction and regulation of immunity in the TME by presenting tumor-associated antigens on major histocompatibility complex (MHC) molecules and supporting T cell responses via producing costimulatory factors. Bearing this unique traits and being able to pass them onto produced exosomes (Dex) or being activated by targeted exosomes gives them a prominent spot in recent cancer therapy studies [78]. Novel breast cancer derived exosomes HELA-Exos were successfully used in triple-negative breast cancer (TNBC) mouse xenograft model and patient-derived tumor organoids, causing activation of DCs in tumor site and subsequent tumor reactive CD8^+^T cell infiltration, causing favorable tumor inhibition [9]. Furthermore, same principle and the exosomal ability to cross blood–brain barrier was exploited by chimeric STING exosomes, which proved to be effective in glioblastoma [79]. Recent research presented universal strategy for Dex nano vaccines production with high efficiency in hepatocellular carcinoma, being able to accommodate the approach for different tumors swiftly [8]. Ongoing Dex clinical trials and shift to multimodal delivery systems along with satisfactory response rates, favorable safety profile and unique abilities of Dex hold a great potential to be highly effective, available, and personalized cancer therapy tools once obstacles like scaling up the production or separation and purification hurdles are overcome.

### T cell-derived exosomes

**Fig. 2:**
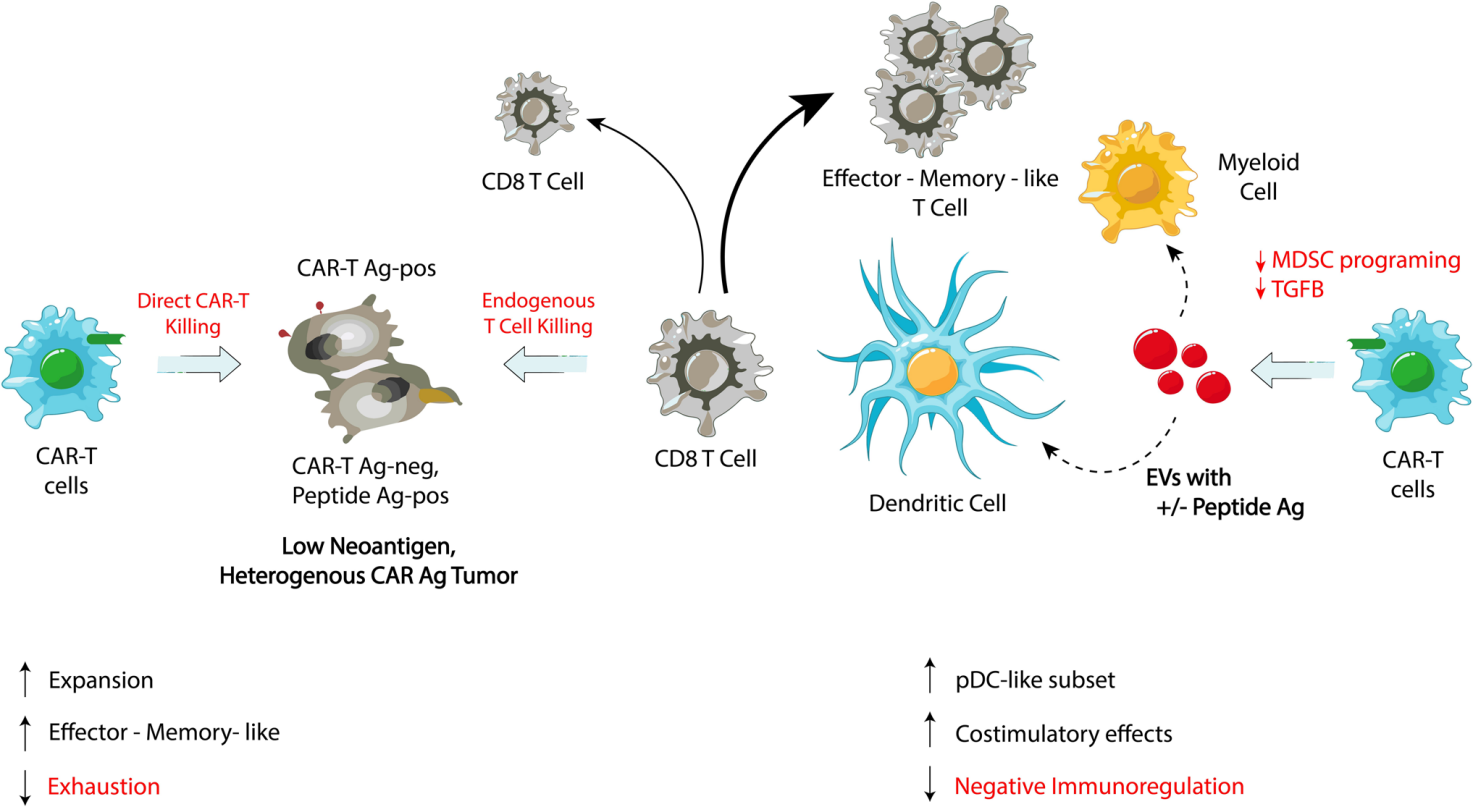
CAR-T cells and mechanisms of action in solid cancer. CAR-T cells mediate direct cytotoxic killing of CAR-T antigen-positive (Ag-pos) tumor cells and promote endogenous CD8^+^ T cell killing of antigen-negative (Ag-neg) tumor cells. The presence of low expressing neoantigens and heterogeneous CAR-T antigens in the tumor microenvironment poses challenges, leading to differential immune responses. CAR-T cells can also interact with dendritic cells and myeloid cells, influencing immune regulation. Extracellular vesicles (EVs) carrying peptide antigens (Ag^+/−^) play a role in modulating these interactions, impacting myeloid-derived suppressor cell (MDSC) programming and TGF-β signaling. Enhanced effector memory-like T cell expansion and reduced exhaustion are observed, but negative immunoregulation mechanisms may limit efficacy

T lymphocytes are key part of adaptive immunity comprising of different subtypes with different roles as CD4^+^ helper cells with their important subclass of CD4^+^CD25^+^ regulatory T cells (Tregs), interacting with other immune cells via produced cytokines or CD8^+^ cytotoxic cells responsible for direct killing of recognized harmful cells like tumor cells. Recent study marked miR-215-5p and miR-375 micro RNAs, found to be highly expressed in CD4^+^ derived EVs after IL-2 stimulation, as capable of dampening cancer cell growth via direct increase of CD8^+^ T cell cytotoxic activity [80]. CD4^+^ derived EVs have also successfully primed macrophages to elevate STING activation, implying that TME macrophages can be reprogrammed [81]. Recently, Vδ2-T cell-derived exosomes triggered apoptosis in Epstein-Barr virus (EBV)-associated gastric carcinoma cells, in Rag2^−/−γc−/−^ mice [82]. Another study described that CD45RO^−^CD8^+^ T cells exosomes express miR-765 which hampered epithelial-mesenchymal transition (EMT) in estrogen driven endometrial cancer [83]. Recently, Jurkat T cell-derived expressing interleukin 2 (IL2) EVs (IL2-sEVs) stimulated CD8^+^ T cells and simultaneously suppressed PD-L1 expression in melanoma cells in immunocompetent mice, exploiting several miRNAs, making it potent therapy capable of immune and cancer cells regulation on miRNA level [84]. Chimeric antigen receptor T cells (CAR-T) are genetically modified T cells expressing chimeric antigen receptors, which gives them unique homing abilities, which are depicted in Fig. 2. The development of exosomes capable of T cell activation and simultaneous delivery of CAR mRNAs was described in a recently published study [85]. But CAR-T cells are also capable to produce exosomes themselves. Exploiting that, CAR-T derived exosomes carrying paclitaxel were tested in mouse xenograft models when inhaled. The exosomes smoothly reached the lungs and halted tumor growth by increasing the number of CD8^+^ T cells and IFN-γ / TNF-α levels in the TME, with no systemic toxicity triggered [86]. Subsequent study introduced hybrid CAR-T derived exosomes fused with lung targeted liposomes and loaded with paclitaxel as a cargo. This combination proved targeted delivery with significant accumulation in the lung tumor cells and reversing immunosuppressive effect of the TME [87]. Moreover, platform utilizing engineered tDC derived Exo-OVA-aCD3/aEGFR was able to successfully preserve the CAR components and co-stimulating molecules needed to T cell activation and bolstered T cells affinity to cancer cells via simultaneously targeting two antigens at the same time. Subsequent in vivo studies in B16-OVA mice demonstrated that the platform showed great antitumor activity and inhibition of tumor recurrence and metastasis. The synergistic effect of the platform used with anti-PD-L1 therapy was also confirmed [88]. These new encouraging data again point out the shift to powerful and easy-delivery systems precisely targeting cancer cells with little or no side effects in comparison with conventionally available therapeutic approaches and might become widespread readily available and effective cancer therapy. To achieve this, hurdles like securing stable and reliable production or effective cargo packaging and prolonged shelf life must be addressed.

### B cell-derived exosomes

B cells, representing the other part of the adaptive immune system complementing T cells, have unique ability to produce specific targeted antibodies when activated, and those can be packed into or expressed on B cell-derived exosomes or EVs. Successful separation of large and small B cell-derived EVs and assessment of their characteristics, activity and cargo revealed that primary B cells EVs display monomeric immunoglobulin M (IgM) on their surface and also carry encapsulated monomeric IgM, being distinct from secreted pentameric IgM. The research data showed that EV IgM bind antigen specifically, and can be incorporated intracellularly into sensitive target cells, which represents an independent and unique antibody distribution system [89]. Lately, transgenic mice were used to unveil that germinal center B cells are capable of IG class switching to express IgG when induced, leading their progeny memory B cells and plasma B cells, to mass production of circulating antigen-specific IgG^+^ EVs. Subsequently, further research on a mouse model of influenza, revealed that IgG^+^ EVs specific for the influenza hemagglutinin antigen effectively protect against the infection. In addition, crossing the B cell Cre driver EV reporter mice onto the Nba2 lupus prone strain generated elevation in circulating EVs expressing surface IgG against nuclear antigens linked to autoimmunity [90].

### NK cell-derived exosomes

Natural killer (NK) cells represent one of major subsets of lymphocytes besides T and B cells, possess the tumor cell killing ability, and modulate immune responses. Recent study evaluated the miRNAs of NK exosomes and described highly expressed subset of miRNAs, amongst which, high expression of let-7b-5p miRNA was observed and lead to cell proliferation arrest in pancreatic cancer cells, representing a novel mechanism by which NK cells hamper tumor growth. However, both cytolytic activity and miRNA content of NK exosomes were weakened upon co-culture with pancreatic cancer cells [91]. Furthermore, NK derived exosomes loaded with miR-30c, upregulated IFN-γ and TNF-α secretion and bolstered the cytotoxicity of NK cells to lung cancer cells, thus stopping tumor growth in vivo [92]. Another recent study revealed that EVs derived from human NK-92 cells stimulated with IL-15 and IL-21 exert enhanced cytotoxicity against tumor cells along with granzyme B and H enrichment, and surprisingly are absorbed by target cells via macropinocytosis [93]. Another research study described human memory-like NK EVs (mNK-sEV) cocultured with IL-12, IL-15, and IL-18, possessing great cytokine secretory ability, capable of entering cancer cells through macropinocytosis and triggering caspase-dependent pathway apoptosis. Those NK EVs achieved greater effectivity in comparison with conventional NK EVs, exerting upregulated level of granulysin and higher tumor affinity in mouse gastric tumor models [94]. Recently, a novel biomimetic platform exploiting a combination of chimeric antigen receptor-natural killer (CAR-NK) cell-derived exosomes (ExoCAR), and a Micelle nano bomb named ExoCAR/T7@Micelle was tested, successfully crossed the blood–brain barrier and selectively targeted HER2^+^ breast cancer cell brain metastases releasing cargo at desired sites leading to a robust antitumor response in vivo, with a significant extension in survival time, without any side effects [95].

### CAF derived exosomes

Cancer-associated fibroblasts (CAFs) represent a key stromal cell population in the TME, thus having crucial role in cancer progression and therapy. Research studies suggested that CAF derived exosomes are also important part of immune response, drug resistance, and tumor progression. Recent study reported a CAF reprograming strategy turning them to effective drug depot in pancreatic cancer, using a hybrid drug delivery system PI/JGC/L-A consisting of a pIL-12-loaded polymeric core (PI) plus JQ1 thienotriazolodiazepine and gemcitabine elaidate coloaded liposomal shell (JGC/L-A). When this system reaches the CAF barrier it stimulates the cells to produce gemcitabine loaded exosomes and upregulates IL-12 secretion, mediating potent drug delivery to the tumor site, bolstering antitumor immunity, and exerting significant antitumor effects [96]. Another study revealed that CAF derived exosomes expressing CD9 and CD63 significantly diminished proliferative capacity of myeloma cell lines and lead to improved 5-year disease-free survival in patients bearing CAF derived CD9 positive exosomes compared to CAF derived CD9 negative patients [97].

### Tumor derived exosomes

Tumor-derived exosomes (TDEs) exhibit dual roles in cancer biology, serving as both facilitators of tumor progression and potential therapeutic agents, leading to ongoing debates in the scientific community. On one hand, TDEs contribute to cancer progression by promoting metastasis, modulating the TME, and suppressing immune responses. For instance, they can carry immunosuppressive molecules that inhibit the activity of cytotoxic T cells and NK cells, thereby allowing tumor cells to evade immune surveillance. Additionally, TDEs can facilitate the formation of pre-metastatic niches by altering the local environment of distant organs, making them more favorable to tumor cell colonization [98]. Conversely, TDEs have been explored for their therapeutic potential. Their ability to carry tumor antigens makes them eligible candidates for cancer vaccine development, aiming to stimulate anti-tumor immune responses. Moreover, TDEs can be engineered to deliver therapeutic agents directly to tumor cells, leveraging their natural targeting capabilities. However, the inherent tumor-promoting properties of TDEs raise concerns about their safety and efficacy as therapeutic tools. Strategies to mitigate these risks include modifying exosomes to enhance their immunostimulatory properties while reducing their immunosuppressive effects have been introduced [99]. These conflicting findings underscore the complexity of TDEs’ roles in cancer and highlight the need for a nuanced approach in their application. While they hold promise for therapeutic interventions, careful consideration of their dual nature is essential to ensure that their utilization does not inadvertently promote tumor progression.

### Exosome-based treatments and achievements

As shown in Table 3, exosome-based therapies have demonstrated significant potential in cancer treatment by enhancing drug delivery, modulating immune responses, and overcoming drug resistance. The exosome-mediated delivery of chemotherapeutic agents, miRNAs, and other therapeutic cargo often in combination to achieve synergistic effects has yielded notable results in glioma, colorectal, breast, pancreatic, lung, and prostate cancers [33, 39, 44, 47, 58, 68]. These findings underscore the versatility of exosome-based approaches, with applications ranging from targeted drug delivery to immune modulation and tumor suppression. While promising, further clinical validation is required to optimize efficacy, safety, and scalability for widespread clinical application.

## Hurdles & new horizons for immune cell-derived exosomes in solid cancer treatment

Exosomes have gained significant attention in cancer therapy due to their potential to enhance treatment efficacy through synergistic mechanisms. These extracellular vesicles, naturally released by cells, can be engineered to deliver a combination of therapeutic agents directly to tumor sites, improving therapeutic outcomes. A notable example involves the engineering of blood-derived exosomes to co-deliver doxorubicin and a microRNA-21 inhibitor. This dual delivery system demonstrated enhanced tumor targeting and significant inhibition of tumor growth in mouse models, highlighting the potential of exosome-mediated combination therapies in oncology [100]. Similarly, in colorectal cancer, engineered exosomes have been utilized to co-deliver 5-fluorouracil (5-FU) and a miR-21 inhibitor, effectively reversing drug resistance and significantly inhibiting tumor growth in mouse models [48]. These studies illustrate a recurring theme: by integrating chemotherapeutic agents with miRNA modulators, exosome-based systems address tumor heterogeneity and drug resistance more effectively than single-agent therapies. In triple-negative breast cancer (TNBC), engineered exosomes co-delivered doxorubicin and hydrophobically modified microRNA-159, enhancing drug uptake and demonstrating synergistic therapeutic effects in vitro and in vivo [39]. Similarly, exosome-sheathed porous silica nanoparticles were developed for the co-delivery of 3,3′-diindolylmethane and doxorubicin, effectively attenuating cancer stem cell-driven epithelial-mesenchymal transition (EMT) and improving therapeutic outcomes [40]. These examples underscore the promise of exosome-based combination therapies in overcoming the complexities of cancer biology, such as EMT, drug resistance, and tumor heterogeneity. However, these findings primarily derive from preclinical studies, necessitating further validation in clinical trials.

Despite their potential, the heterogeneity of exosomes remains a significant challenge. Cells secrete diverse exosome populations, even within the same cell type, leading to variability in molecular composition and targeting properties. This heterogeneity complicates dosage standardization, therapeutic delivery, and clinical applications. Molecular mechanisms governing exosome biogenesis, such as the ESCRT-dependent and -independent pathways, contribute to this variability, yet these mechanisms remain poorly understood [101]. A deeper understanding of the signaling pathways involved in exosome sorting, cargo loading, and targeting is essential. For instance, miRNAs delivered by exosomes often modulate key pathways such as PI3K/AKT, WNT/β-catenin, or JAK/STAT, but the precise mechanisms vary across studies and cancer types [9, 102]. Future research should focus on identifying exosome subpopulations tailored for specific therapeutic purposes, while also mitigating unintended effects, such as tumor promotion or off-target immune activation.

The standardization of exosome isolation and purification also presents significant technical hurdles. Traditional methods such as ultracentrifugation and size-exclusion chromatography (SEC) are widely used but yield heterogeneous exosome populations with variable functionality. Emerging techniques, including microfluidic devices and tangential flow filtration, show promise in improving purity and scalability [103]. For example, density gradients in ultracentrifugation enhance purity by reducing co-isolation of contaminants [104], while SEC combined with ultrafiltration addresses sample dilution [105]. Immunoaffinity techniques offer exceptional specificity by targeting exosome surface markers but are limited by high costs and scalability challenges [106]. Microfluidic platforms enable rapid, high-purity isolation with minimal sample requirements, but issues such as low throughput and lack of standardization persist [107]. Table 4 summarizes the advantages and limitations of these techniques. Developing scalable, cost-efficient, and reproducible isolation protocols is critical for advancing exosome-based therapies [108].

**Table 4:** Pros and cons of isolation and purification techniques of exosomes

Method	Principle	Pros	Cons	Reference
Ultracentrifugation-Based Techniques	Ultracentrifugation is the gold standard for exosome isolation, separating exosomes based on size, density, and shape through sequential high-speed centrifugation	• Capable of processing large sample volumes • Does not require specialized reagents	• Time-consuming and labor intensive • Requires expensive equipment • May co-isolate contaminants (proteins, lipoproteins) • High-speed centrifugation may damage exosomes	[109]
Size-Based Techniques	Isolate exosomes based on size differences compared to other particles Common approaches include ultrafiltration and size-exclusion chromatography (SEC)	• Rapid and straightforward • Does not require special equipment • Provides high-purity exosomes • Preserves exosome integrity and biological activity • Offers excellent reproducibility	• Prone to membrane clogging and vesicle trapping, reducing efficiency • Potential loss of exosomes due to adhesion to the membrane • Not easily scalable • Long processing times	[110]
Immunoaffinity Capture-Based Techniques	Uses antibodies to bind to exosomal surface proteins, allowing selective isolation of specific exosome subpopulations	• Enables high-purity isolation of specific exosome subsets • Facilitates detailed studies on particular exosome functions	• High reagent costs • Limited by antibody specificity • Typically yields lower exosome quantities • May not capture all exosome subtypes due to variability in surface protein expression	[111]
Precipitation-Based Techniques	Alters the solubility of exosomes using polymers, causing precipitation out of solution	• Simple and easy to perform • Does not require specialized equipment • Scalable for larger sample volumes	• Co-precipitation of contaminants (proteins, extracellular vesicles) • Requires additional purification steps • Yield and purity vary based on sample type and conditions	[112]
Microfluidics-Based Techniques	Isolate exosomes based on size, density, and immunoaffinity using microscale devices	• Fast and efficient processing • Low sample and reagent consumption • Potential for integration with downstream analytical techniques	• Limited scalability for large sample volumes • Challenges in standardization and clinical validation • Some devices require complex fabrication and operation	[113]

Immune cell-derived exosomes offer unique opportunities for cancer therapy due to their ability to modulate immune responses. For instance, macrophage-derived exosomes evade phagocytosis, enhancing stability and drug delivery efficacy, while DC-derived exosomes facilitate antigen presentation and tumor rejection [114]. However, the TME often dampens T cell responses, limiting the efficacy of exosome-based immunotherapies. Tumor-derived exosomes, carrying tumor-associated antigens and MHC class I molecules, show potential in activating immune responses, but their use poses risks of tumor progression or metastasis [102]. Selecting appropriate exosome sources and optimizing their engineering to enhance immune responses without exacerbating tumorigenesis remain key challenges. Non-tumor sources, such as red blood cell (RBC)-derived exosomes, present a safer alternative. Their enucleated nature reduces risks of horizontal gene transfer and immunogenic responses, offering a practical platform for therapeutic applications [115]. Similarly, agricultural sources such as milk-derived or plant-derived exosomes are cost-effective and scalable but lack the immune-boosting capabilities required for certain therapies [114]. These sources may complement other approaches, especially in combination therapies where immune activation is less critical.

Emerging strategies to improve the clinical translation of exosome-based therapies include advanced administration methods, such as PEGylation to extend circulation time, local injections for site-specific delivery, and intranasal delivery to bypass the blood–brain barrier [116]. Additionally, innovative platforms such as hydrogels for sustained release and combination therapies have demonstrated enhanced therapeutic responses in preclinical models [117]. Despite these advances, challenges remain in maintaining the stability and functionality of exosomes in vivo. Preclinical studies, such as those combining cytostatic drugs with exosomes derived from crab hemolymph, underscore the untapped potential of exosome therapies but also highlight the need for broader validation [118].

## Conclusion

Exosomes hold immense promise in the realm of cancer immunotherapy, particularly for solid tumors where conventional treatments often fall short. Their ability to transport biologically active cargo across cellular barriers, modulate immune responses, and be engineered for targeted therapy positions them as versatile tools for next-generation cancer treatments. Recent advancements in engineered exosomes have demonstrated potential to enhance drug delivery, overcome drug resistance, and improve tumor specificity. Moreover, emerging strategies, such as the combination of exosomes with immune checkpoint inhibitors and RNA-based therapeutics, offer synergistic effects that could transform clinical outcomes.

Despite these advancements, significant challenges remain, including exosome heterogeneity, standardization of isolation and purification techniques, and scalability for clinical use. Addressing these issues will require concentrated efforts to optimize exosome engineering, develop robust manufacturing protocols, and improve our understanding of their mechanisms of action. As research progresses, the integration of exosome-based therapies into current cancer treatment protocols has the potential to revolutionize oncology, offering safer and more effective options for patients with solid tumors.

## Data Availability

No datasets were generated or analysed during the current study.

## Abbreviations

APC: Antigen-Presenting Cell
BBB: Blood–Brain Barrier
CAF: Cancer-Associated Fibroblast
CAR-T: Chimeric Antigen Receptor T Cell
CCR5: Chemokine Receptor for Immune Cell Migration
CD: Cluster of Differentiation
CD25^+^: Regulatory T Cell Marker
CD3: T Cell Co-Receptor
CD4^+^: Helper T Cell Subtype
CD45RO: Memory T Cell Marker
CD63: Exosome Marker
CD8^+^: Cytotoxic T Cell Subtype
CD86: Co-Stimulatory Molecule in APCs
CD9: Tetraspanin Marker for Exosomes
CRISPR: Clustered Regularly Interspaced Short Palindromic Repeats
DC: Dendritic Cell
DEX: Dendritic Cell-Derived Exosomes
Dex-NP: Dendritic Cell Exosome-Based Nanoparticle
ECM: Extracellular Matrix
EGFR: Epidermal Growth Factor Receptor
ELANE: Human Neutrophil Elastase
EMT: Epithelial-Mesenchymal Transition
EV: Extracellular Vesicle
ExoCAR: CAR-NK Cell-Derived Exosomes
FA-ExoPAC: Folic Acid-Modified Exosome-Loaded Paclitaxel
FasL: Fas Ligand
GBM: Glioblastoma
GM-CSF: Granulocyte–Macrophage Colony-Stimulating Factor
HCC: Hepatocellular Carcinoma
HMGN1: High-Mobility Group Nucleosome-Binding Protein 1
ICIs: Immune Checkpoint Inhibitors
IFN-γ: Interferon-Gamma
IL: Interleukin
IL-12: Interleukin-12
IL-15: Interleukin-15
IL-18: Interleukin-18
IL-2: Interleukin-2
ILVs: Intraluminal Vesicles
MDSC: Myeloid-Derived Suppressor Cell
MHC: Major Histocompatibility Complex
MHC-I: Major Histocompatibility Complex Class I
MHC-II: Major Histocompatibility Complex Class II
miRNA: MicroRNA
MISEV: Minimal Information for Studies of Extracellular Vesicles
mRNA: Messenger RNA
MVB: Multivesicular Body
MsEV: Mesenchymal Stem Cell-Derived Extracellular Vesicle
NCT: National Clinical Trial (Identifier for Clinical Trials)
NK: Natural Killer (Cell)
OVA: Ovalbumin (Model Antigen)
OVs: Oncolytic Viruses
PD-1: Programmed Death 1
PD-L1: Programmed Death-Ligand 1
PEGylation: Polyethylene Glycol Conjugation
PI3K/AKT: Phosphoinositide 3-Kinase/Protein Kinase B Pathway
PTEN: Phosphatase and Tensin Homolog
sEV: Small Extracellular Vesicle
siRNA: Small Interfering RNA
STAT: Signal Transducer and Activator of Transcription
STING: Stimulator of Interferon Genes
TDE: Tumor-Derived Exosome
TLR3: Toll-Like Receptor 3
TME: Tumor Microenvironment
TNBC: Triple-Negative Breast Cancer
TNF: Tumor Necrosis Factor
TRAIL: TNF-Related Apoptosis-Inducing Ligand
α-LA: Alpha-Lactalbumin
5-FU: 5-Fluorouracil
